# Verbal Learning as a predictor of risks of accidents in elderly drivers

**DOI:** 10.1590/0004-282X-ANP-2021-0054

**Published:** 2021-12-31

**Authors:** Adriana Machado Vasques, Wyllians Vendramini Borelli, Márcio Sarroglia Pinho, Mirna Wetters Portuguez

**Affiliations:** 1 Brain Institute of Rio Grande do Sul, Pontifícia Universidade Católica do Rio Grande do Sul, Porto Alegre RS, Brazil. Brain Institute of Rio Grande do Sul Pontifícia Universidade Católica do Rio Grande do Sul Porto Alegre RS Brazil; 2 Instituto de Geriatria e Gerontologia, Pontifícia Universidade Católica do Rio Grande do Sul, Porto Alegre RS, Brazil. Instituto de Geriatria e Gerontologia Pontifícia Universidade Católica do Rio Grande do Sul Porto Alegre RS Brazil; 3 Faculdade de Medicina, Pontifícia Universidade Católica do Rio Grande do Sul, Porto Alegre RS, Brazil. Faculdade de Medicina Pontifícia Universidade Católica do Rio Grande do Sul Porto Alegre RS Brazil; 4 Escola Politécnica, Pontifícia Universidade Católica do Rio Grande do Sul, Porto Alegre RS, Brazil. Escola Politécnica Pontifícia Universidade Católica do Rio Grande do Sul Porto Alegre RS Brazil

**Keywords:** Automobile Driving, Memory, Risk Management, Aged, Condução de Veículo, Memória, Gestão de Riscos, Idoso

## Abstract

**Background:**

Age-related cognitive decline impacts cognitive abilities essential for driving.

**Objective:**

We aimed to measure main cognitive functions associated with a high number of traffic violations in different driving settings.

**Methods:**

Thirty-four elderly individuals, aged between 65 and 90 years, were evaluated with a driving simulator in four different settings (Intersection, Overtaking, Rain, and Malfunction tasks) and underwent a battery of cognitive tests, including memory, attention, visuospatial, and cognitive screening tests. Individuals were divided into two groups: High-risk driving (HR, top 20% of penalty points) and normal-risk driving (NR). Non-parametric group comparison and regression analysis were performed.

**Results:**

The HR group showed higher total driving penalty score compared to the NR group (median=29, range= 9-44 vs. median=61, range= 47-97, p<0.001). The HR group showed higher penalty scores in the Intersection task (p<0.001) and the Overtaking and Rain tasks (p<0.05 both). The verbal learning score was significantly lower in the HR group (median=33, range=12-57) compared with the NR group (median=38, range=23-57, p<0.05), and it was observed that this score had the best predictive value for worse driving performance in the regression model. General cognitive screening tests (Mini-Mental State Examination and Addenbrooke's Cognitive Evaluation) were similar between the groups (p>0.05), with a small effect size (Cohen’s d=0.3 both).

**Conclusion:**

The verbal learning score may be a better predictor of driving risk than cognitive screening tests. High-risk drivers also showed significantly higher traffic driving penalty scores in the Intersection, Overtaking, and Rain tests.

## RESUMO

**Antecedentes:**

O declínio cognitivo relacionado à idade impacta as habilidades cognitivas essenciais para direção.

**Objetivos:**

Nosso objetivo foi medir as funções cognitivas associadas ao alto número de violações de trânsito em diferentes contextos de direção.

**Métodos:**

Trinta e quatro idosos entre 65 e 90 anos foram avaliados em simulador de direção em quatro diferentes contextos (Travessia, Ultrapassagem, Chuva e Mal-funcionamento) e realizaram uma série de testes cognitivos, incluindo memória, atenção, visuoespacial e rastreamento. Indivíduos foram então divididos em dois grupos: Alto Risco de condução (HR, top 20% de pontos de penalidades de condução), e Risco Normal (NR). Comparações não-paramétricas e análise de regressão foram realizadas.

**Resultados:**

O grupo HR mostrou aumento no escore total de penalidades de condução quando comparado com o grupo NR (mediana=29, limites=9-44 vs. mediana=61, limites=47-97, p<0.001). O grupo HR mostrou maiores escores de penalidade na tarefa de Travessia (p<0.001), Ultrapassagem e Chuva (p<0.05 ambos). O escore de aprendizado verbal foi significativamente menor no grupo HR (mediana=33, limite=12-57) comparado com o grupo NR (mediana=38, limite=23-57, p<0.05), e foi observado que este escore foi o melhor preditor de pior performance de condução no modelo de regressão. Testes de rastreio cognitivo (Mini-exame do estado mental e Avaliação Cognitiva de Addenbroke) foram similar entre os grupos (p>0.05), com pequena magnitude de efeito (Cohen’s d=0.3).

**Conclusões:**

O escore de aprendizado verbal pode ser o melhor preditor de risco de condução do que os testes de rastreio cognitivos. Motoristas de alto risco também mostraram maior escores de penalidade de trânsito nos testes de Travessia, Ultrapassagem e Chuva.

## INTRODUCTION

Traffic accidents are a major cause of death in all age groups^1^. There is a growing number of individuals driving cars in urban areas, especially older adults^2^, and with the constant increase in the geriatric population, the number of older adults obtaining a driver's license has increased^3^. However, age-related cognitive decline affects driving abilities and may increase the number of traffic violations and accidents^4^. 

Driving plays an important role in social integration and promotes psychological and physical well-being. There is a clear association between driving cessation and impact on autonomy and quality of life. Driving cessation in elderly leads to an important shift in productive engagement and to higher rates of depressive symptoms^5^^,^^6^. The transition to a stage of inability to drive may be inevitable with cognitive decline, but it is not crlearly defined in older adults without dementia^7^.

As driving a vehicle is a highly complex task, several cognitive skills must be integrated simultaneously. Assessing driving capacity requires a broad evaluation of both qualitative and quantitative measures, including cognitive, psychological, and emotional factors. Some earlier studies did not find any clear association between a cognitive measure and driving capacity^8^. Safe driving requires adequate functioning of attentional and visuospatial skills, which are domains that typically decline with aging. Furthermore, older adults have longer reaction times^9^ and tend to make more safety errors while driving^10^. Visual-spatial abilities and their integration with motor tasks are more vulnerable to the aging process, and therefore, severely affect the driving skills of older adults.

Driving simulators have been widely used for multiple neurological conditions^11^. There are some strengths and weaknesses in assessing driving abilities with a simulator^12^. A driving simulator, most commonly used for improving driving performance in older adults, is also useful for attention skills^13^. Older drivers in particular may benefit from a simulator setting because it is an efficient and valid training instrument for multiple cognitive abilities^12^ and provides a safe environment. Besides, it is also useful in generating highly accurate research data in studies involving neurocognitive abilities and their relationship to drivers. On the other hand, a simulator may not correspond to the reality of driving, apart from the motion sickness associated with virtual reality^14^^,^^15^.

Impaired driving due to an underlying neurological condition may pose a risk to society. There are many studies showing a higher incidence of traffic violations in individuals with dementia^16^. Patients diagnosed with Parkinson’s or Alzheimer’s disease show significantly high executive difficulties and reduced operational level in driving performances^11^^,^^15^. Brain amyloid burden is also associated with a significantly higher crash risk, even in asymptomatic individuals^17^^,^^18^. Furthermore, even healthy older adults are vulnerable to severe injuries caused by traffic accidents, with slower recovery rate than younger individuals^19^. 

In this context, the evaluation of asymptomatic healthy older adults is fundamental. A higher risk of collision exists for many individuals without known neurological condition. We investigated the relationships between some commonly used neuropsychological tests in older adults at high risk for driving violations and in different driving situations.

## METHODS

### Participants

Thirty-three individuals aged between 65 and 90 years (median age = 71) with a valid national driver’s license and still driving were included. Nine individuals from the initial sample were excluded because of “simulator sickness”, which is a type of motion sickness related with virtual reality ^14^. All participants gave written informed consent and had have no clinical evidence of neurological or psychiatric disease (Beck Anxiety Inventory < 16 and Geriatric Depression Scale < 6)^20^^,^^21^ and preserved activities of daily living. They were recruited from the general population that volunteered or entered the Driving Center for any reason. All had normal or corrected-to-normal vision and none reported having a hearing problem.

### Road driving simulator

Participants were asked to drive on a road driving simulator (Auto SmartSim, Esteio, Brazil, 2013) in four different situations. Following a previous study^22^ and based on an experienced driving instructor, four driving situations were selected for this study. These scenarios were chosen based on frequent traffic violations by older adults^23^^,^^24^. Initially, they performed a training session of five minutes without traffic. At first, in the *Intersection* scenario, the driver had to cover a guided route with pedestrian lanes, traffic lights, and signaled intersections where people and cyclists cross the road. In the second scenario, *Overtaking*, the driver must perform a safety overtaking maneuver with a car. In the third scenario, *Rain*, the driver must control the vehicle during rain and fog. Fourth, *Malfunction*, the driver must detect an electrical or mechanical malfunction of the engine and give and appropriate signal to stop. The duration of each test varied (range=12-30 min).

### Penalty scores

Traffic violations were classified according to the standards of the Brazilian Law, which is based on the Vienna Convention on Road Traffic^25^. Participants received penalty points according to the level of the penalty (Table 1). Scores were automatically attributed according to traffic violations during the trips and manually checked in the meantime.

**Table 1.** Description of International Driving Law and correspondence to driving scores.

Driving penalty (points)	Description of traffic violations
Mild (1)	Touching the clutch pedal while driving
Moderate (2)	Turning off the car while driving; sudden stopping; driving with the handbrake pulled; incorrect upshifting; driving in neutral
Severe (3)	Turning with signaling errors
Most severe (4)	Colliding; Driving above the speed limit; Passing a red traffic light

There were four types of errors, with scores from 1-4 according to the severity of traffic violation. Traffic violation score was the total number of errors performed during the four tasks. 

According to a previously described procedure^26^, individuals were then separated in two subgroups, High-risk (HR) and Normal-risk Driving (NR) by the mean values of the total driving penalty score. The HR group presented significantly higher driving penalties in the total driving penalty score when compared to the NR group (p<0.001). This method successfully classified individuals that performed the top 20% driving penalty scores (>0.5 SD of total mean) of the sample into the high-risk group.

### Neuropsychological evaluation

Soon after the driving simulator, all participants underwent cognitive measurement on relevant domains for driving^8^, according to the availability of cultural validation of the tests. The battery included the Mini-Mental State Examination (MMSE), the Digit Symbol, from the WAIS III^27^, Trail Making Test A (TMTA) and B (TMTB)^28^, Addenbrooke’s Cognitive Evaluation-Revised (ACE)^29^, the Rey Auditory-Verbal Learning Test (RAVLT)^30^, both learning and delayed-recall scores, and the Category Fluency Test with animals (CFT)^31^. We also performed the Test of Divided Attention (TDA)^32^, commonly used for periodic driving evaluation in Brazil. This test evaluates an individual's ability to search and find one different stimulus, randomly distributed among 400 symbols. The total number of symbols correctly pointed in four minutes is recorded. 

### Statistical analysis

The sample normality was assessed with the Kolmogorov-Smirnov test. Spearman’s correlation was used to examine the relationship between age, education, and driving penalty score. Wilcoxon’s sum-rank test was used for the comparison of the cognitive tests and groups. We also evaluated the effect size of cognitive tests using Cohen’s d value. 

A stepwise regression model was run to evaluate which cognitive measure score was most strongly associated with the driving penalty score of each test. We determined the adjusted R-squared and considered it to be statistically significant when p-values were less than 0.05. 

All statistical analyses were performed using R Studio v1.0.136.

## RESULTS

The characteristics of the sample are described in Table 2. There was no gender discrepancy among the driving risk groups (p>0.05). Age was moderately correlated with the Intersection score (r=0.48, p=0.004) and total driving penalty score (r=0.37, p=0.03). The years of education were inversely correlated to the total driving penalty score (r=-0.39, p=0.02).

**Table 2.** Demographic characteristics of the sample.

	Driving risk		
	Normal (n= 27)	High (n= 7)	Total sample
Age, median (range)	70 (65-85)	73 (65-90)	71 (65-90)
Years of education, median (range)	12 (7-18)	11 (8-15)	11 (7-18)
Years of driving, median (range)	44 (20-65)	49 (34-56)	44 (20-65)
Females	9	3	12
MMSE, median (range)	28 (23-30)	28 (21-30)	28 (21-30)
Driving score, median (range)	29 (9-44)	61 (47-97)^a^	30.5 (9-97)
Intersection test	10 (2-33)	38 (6-49) ^a^	14 (2-49)
Overtaking test	4 (0-12)	6 (0-36)*	4 (0-36)
Rain test	4 (0-21)	12 (6-26)*	6 (0-26)
Malfunction test	4 (1-27)	5 (0-18)	4 (0-27)
Cognitive tests, median (range)			
ACE	90.5 (72-98)	91 (68-94)	91 (68-98)
Digit Symbol	49 (28-89)	33 (12-57)	49 (12-89)
TDA	75.5 (6-172)	55 (0-124)	69.5 (0-172)
TMT-A	72 (36-115)	81 (45-101)	74.5 (36-115)
TMT-B	135 (62-279)	199 (95-300)	135 (62-300)
RAVLT-Sum	38 (23-57)	33 (20-38)*	37.5 (20-57)
RAVLT-Delayed recall	7 (2-14)	6 (0-8)	6.5 (0-14)
CFT	16.5 (3-28)	14 (8-19)	15.5 (3-28)

MMSE: Mini-Mental State Examination; ACE: Addenbrooke’s Cognitive Evaluation; DS: Digit Symbol; TDA: Test of Divided Attention; TMT-A: Trail Making Test A; TMT-B: Trail Making Test B; RAVLT-Sum: Rey auditory-verbal learning test, the sum from A1 to A5 lists; RAVLT-A7: Rey auditory-verbal learning test, delayed-recall list; CFT: Category Fluency Test, Animals; *p<0.05; ^a^p<0.001.

The HR group showed higher driving penalty scores in the Intersection, rain, and overtaking tests compared to the NR group. Malfunction test scores were similar between both groups and presented a moderate effect size (Cohen’s d=-0.59). The HR group showed a significantly lower learning score in the RAVLT than the NR group, but not in the delayed-recall test (p=0.45). The Digit Symbol test (Cohen’s d=0.97) and the learning score of the RAVLT (Cohen’s d=1.006) had the largest effect sizes of performed tests. MMSE and ACE showed small effect sizes (Cohen’s d=0.3 both).

In the regression model, we included the TDA (p=0.02), Digit Symbol test (p=0.002), TMTB (p=0.1), learning score of the RAVLT (p=0.009), delayed-recall of the RAVLT (p=0.09), and visuospatial subtest of the ACE (p=0.02). The results of the regression analysis are shown in Table 3 and the correlation between tests are shown in Table 4. 

**Table 3.** Stepwise regression model adjusted for education.

Tests	R^2^	Regression coefficient		Standardized coefficient	p-values
		B	Standard error b	Beta	
Total driving score	0.568				
(Constant)		139.022	37.711		**0.001**
RAVLT-Sum		-0.912	0.333	-0.4277	**0.007**
Visuospatial		-4.646	2.610	-0.28	0.085
Intersection	0.501				
(Constant)		40	8.32		**<0.001**
RAVLT-Sum		-0.983	0.306	-0.71	**0.002**
RAVLT-A7		1.798	0.920	0.432	0.059
Overtaking	0.616				
(Constant)		59.911	14.013		**<0.001**
Visuospatial		-4.871	1.333	-0.814	**0.002**
TDA		-0.042	0.026	-0.244	0.108
Rain	0.603				
(Constant)		26.726	5.751		**<0.001**
TDA		-0.076	0.028	-0.482	**0.010**
TMT-A		-0.103	0.050	-0.360	0.050
RAVLT-A7		-0.766	0.344	-0.375	**0.033**
Malfunction	0.496				
(Constant)		-47.860	18.881		**0.017**
TMT-A		0.132	0.049	0.490	**0.010**
Attention/Orientation		1.969	0.949	0.362	**0.047**
Memory (ACE)		0.506	0.281	0.298	0.076

RAVLT-Sum: Rey auditory-verbal learning test, the sum from A1 to A5 lists; RAVLT-A7: Rey auditory-verbal learning test, delayed-recall list; TDA: Test of Divided Attention; TMT-A: Trail Making Test A; ACE: Addenbrooke’s Cognitive Evaluation. (P < 0.05 in bold)

**Table 4.** Intercorrelations among cognitive measures and driving tasks.

Measures	1	2	3	4	5	6	I	O	R	M
DS (1)	1									
TDA (2)	0.64	1								
RAVLT-Sum (3)	0.50	0.39	1							
RAVLT-A7 (4)	0.45	0.45	0.71	1						
ACE (5)	0.36	0.57	0.48	0.46	1					
CFT (6)	0.34	0.41	0.50	0.26	0.57	1				
**Driving tasks**										
Intersection (I)	-0.25	-0.14	-0.40	-0.07	-0.20	-0.27	1			
Overtaking (O)	-0.46	-0.40	-0.30	-0.26	-0.26	-0.10	0.23	1		
Rain (R)	-0.34	-0.46	-0.45	-0.44	-0.44	-0.19	0.19	0.54	1	
Malfunction (M)	-0.20	-0.08	0.10	-0.11	0.24	0.13	-0.06	0.03	-0.12	1
Total Score	-0.49	-0.40	-0.48	-0.31	-0.29	-0.23	0.77	0.68	0.59	0.23

DS: Digit Symbol; TDA: Test of Divided Attention; RAVLT-Sum: Rey auditory-verbal learning test, the sum from A1 to A5 lists; RAVLT-A7: Rey auditory-verbal learning test, delayed-recall list; ACE: Addenbrooke’s Cognitive Evaluation; CFT: Category Fluency Test, Animals.

The best predictors of each test were: delayed-recall score of the RAVLT for total driving penalty score (R2 = 0.445); learning and delayed-recall scores of the RAVLT for the Intersection test (R2 = 0.502); the visuospatial subtest of the ACE, the TDA, and the learning score of the RAVLT for the Overtaking test (R2 = 0.617); the TDA, the TMT-A, and the delayed-recall scores for the Rain test (R2 = 0.605); and the TMT-A, the Memory subtest, and the Attention/Orientation subtest of the ACE for the Malfunction test (R2 = 0.506). The Intersection test showed the highest correlation coefficient with the total driving penalty score (r=0.77) when compared with other tests (r=0.68, r=0.59, r=0.23 for Overtaking, Rain, and Malfunction tests, respectively). Furthermore, the RAVLT had an inverse moderate correlation with total driving penalty scores (r= -0.47).

## DISCUSSION

This is the first study from Brazil to evaluate cognitive functions of older adults using a driving simulator. Considering different settings in a driving ability test for older adults is only possible with a reproducible tool such as a simulator. Although Brazil is a country with the a relatively low level of education, our sample had an intermediate level of education, probably because the ability to read and perform basic attention tests are required to obtain a driver’s license. Furthermore, this study will be very useful in the field as the number of older drivers in society is increasing. Life expectancy also increases proportionally, leading to a significant increase in cognitively healthy older adults who still drive and are independent. A driving simulator is essential in identifying older people with a high risk for unsafe driving, thus avoiding any risk associated with an evaluation on the road and accident. In addition, individuals who continue to drive at high risk can be asked to stop driving, avoiding harm on the road.

In this sample, older age was moderately associated with worsening in some cognitive abilities, as well as with the Intersection and total driving penalty scores. The fatal crash rate per mile is increased in drivers over 70 years of age, which confirms our findings^33^. The Mini-Mental State Examination (MMSE) is still the most widely used cognitive screening tool. However, the MMSE score showed a poor correlation with driving performance for both high and low cognitions^34^. In this study, general cognitive evaluation, assessed with the MMSE and the ACE, was not an adequate criterion to discriminate normal from high-risk older drivers. It is possible that these tests are not sensitive in detecting mild to moderate cognitive decline^35^. Besides, our regression analysis confirmed previous studies in which an association between driving and cognition was reported^10^. Total driving score was significantly associated with learning score (Table 3). A single test may not accurately predict driving performance, as this requires a highly complex and synchronous ability between several cognitive domains.

Some specific cognitive tests were associated with HR driving. The learning ability of older drivers, measured by the sum of the five first lists of the RAVLT, was significantly different between the high-risk and normal-risk driving older adults as indicated by a large effect size. Previous studies have shown that learning ability is associated with driving penalty scores but may not be associated with driving skills, but is a potential target for improving driving skills^36^. In this study, the learning score of the RAVLT was moderate and inversely associated with total driving penalty scores. The RAVLT has been found to be a sensitive test for detecting of early signs of cognitive impairment^37^. Among the different tests used in this study, the RAVLT was the only one that could distinguish high-risk older drivers from those at normal risk. Driving assessment may benefit from the RAVLT in the identification of older adults with a high risk for accidents during periodic driver’s license renewal.

Scores for all settings were significantly impaired in the HR group, except the Malfunction test. Perhaps a malfunction forces the driver to stop the car and either ask for help or fixing the problem. The Intersection test was highly correlated with the total driving penalty scores, and learning and delayed-recall abilities (Table 4). This is probably the most commonly performed task for drivers and requires attention to effectively avoid traffic violations and collisions. However, we found that the learning and delayed-recall memory scores, both measured with the RAVLT, were associated with this task. The RAVLT is an important predictor of white matter changes^38^ and other structural brain changes in individuals presenting further cognitive decline^37^^,^^39^. The use of a simulator, however, is also associated with learning skills, which could influence the lower scores on traffic penalty. Besides, the HR group showed an almost three-fold higher score in the Rain test, which corroborates with previous studies on adverse weather conditions^40^. This may be due to the decreased ability to identify traffic signs by older adults with a higher risk during adverse weather conditions with decreased visibility. Other contributing factors, such as the reflex speed and motor responses, are also responsible for an increased crash rate of older drivers^33^. Further studies may corroborate these findings with evaluations on the road. 

Despite our efforts, this study had some limitations that must be discussed. A major limitation is the sensibility of the simulator, which may cause an overrepresentation of scores. Furthermore, our sample consisted only of individuals who accepted the invitation to participate, which may lead to a selection bias toward older adults with better cognitive performance. The sample size was also a factor of limitation, and some results should be replicated to improve external validity. 

In conclusion, high-risk older drivers had lower verbal learning test scores compared with normal-risk older drivers, but the same was not true for general cognition tests. High-risk older drivers also showed significantly higher traffic penalties in Intersection, Overtaking and Rain tests, but not in the Malfunction simulator test compared with the normal-risk older drivers. In addition, the Rey auditory-verbal learning test was the best predictor of safe driving in our regression model.
